# The Congress on School Hygiene

**Published:** 1907-08-17

**Authors:** 


### THE CONGRESS ON SCHOOL HYGIENE.

ALL who have followed the proceedings of the second International Congress on School Hygiene must, we fear, have been reluctantly compelled to admit that in her care for the well-being of her school children, Great Britain stands far behind Germany, Switzerland, the United States, and many other foreign nations. This is due, no doubt, in great part to our general supineness in dealing with all social problems, and for the rest to the many economic difficulties that surround this one in particular. It seems, however, that public interest in the whole question of school hygiene has at length been so far aroused as to claim for it recognition as a matter of vital concern to the nation at large.

The Congress has formulated certain resolutions and recommendations, of which two, concerning the medical inspection of schools and the teaching of hygiene to children, stand out as of prime importance. With regard to the first of these our readers will be aware that numerous, more or less tentative, schemes of inspection have been in operation during the last few years in many of the towns and rural districts of this country, but, inasmuch as these schemes received no official support or recognition from Parliament, it rested with the local authority to institute any system of inspection that seemed best to it, and there has been, therefore, a complete lack of uniformity in the methods employed. The Education (Administrative Provisions) Bill now before Parliament contains a clause requiring the local education authority to provide for the medical inspection of all public elementary schools. This Bill has already passed through committee, and may be expected to become law during the present session. In this event we sincerely hope that those responsible for its administration will spare no effort to establish a thoroughly efficient mechanism of inspection. This may be a matter of some difficulty in view of the varied conditions and the many conflicting interests that will have to be met. There is also a danger that efficiency will be restricted by the pious disinclination of some local authorities to spend money on projects of this nature, as well as by exaggerated ideas of the expenditure required. The cost of the various systems now in force is in most cases covered by a rate of less than $\frac{1}{10}$d.; and, although we look forward to a system somewhat more elaborate than any of these, this figure serves to show that medical inspection involves a very small outlay, which should be more than balanced by a decreased expenditure on the treatment of infectious diseases and by an avoidance of the loss of educational grants.

With regard to the teaching of hygiene, the Congress expressed the opinion "that the principles and practice of hygiene should form part of the education of every citizen," and it recommended that all teachers should become qualified to give instruction in hygiene and physical training. The inclusion of the latter in the school curriculum should lead to the best results. But we are inclined to doubt whether the pupils in elementary schools will derive the full benefit from a course of instruction in hygiene unless this be followed by further instruction in the same subject after they have left school. Hygiene is not an exact science, nor comparable with the other subjects taught in these schools, and to understand thoroughly even its rudiments requires a knowledge of the elements of physiology such as children can scarcely be expected to possess. We believe that the various Polytechnic Institutes and Working Men's Colleges could do a very useful service by giving further instruction of this nature, and their members would be all the more ready to attend such a course in consequence of their previous acquaintance with the subject.

We are pleased to think that the Congress has been a distinguished success in every way. It now remains for all to show their sympathy with its aims and ideals by doing their utmost to put its recommendations into practice. There must first be established an efficient system of medical inspection, and this of itself should do much to improve the health of the community. But we must not be content to let things rest here. We must make it our aim to spread far and wide a knowledge of the fundamental principles of health, to show people how much they can do for themselves by observing a few simple rules and precautions, and to teach them that in this matter everyone has a personal responsibility which he cannot shift on to the shoulders of his neighbours or of the State. As soon as this is accomplished we shall have gone far to find a solution of the urgent problems of infantile mortality, intemperance, and unemployment.

